# Comparative analysis of machine learning algorithms for computer-assisted reporting based on fully automated cross-lingual RadLex mappings

**DOI:** 10.1038/s41598-021-85016-9

**Published:** 2021-03-09

**Authors:** Máté E. Maros, Chang Gyu Cho, Andreas G. Junge, Benedikt Kämpgen, Victor Saase, Fabian Siegel, Frederik Trinkmann, Thomas Ganslandt, Christoph Groden, Holger Wenz

**Affiliations:** 1 Department of Neuroradiology, Medical Faculty Mannheim, Heidelberg University, Theodor-Kutzer-Ufer 1-3, 68137 Mannheim, Germany; 2 Department of Biomedical Informatics at the Center for Preventive Medicine and Digital Health (CPD-BW), Medical Faculty Mannheim, Heidelberg University, Mannheim, Germany; 3 Empolis Information Management GmbH, Kaiserslautern, Germany

**Keywords:** Computational models, Machine learning, Statistical methods, Computational biology and bioinformatics, Data mining, Diagnostic markers, Predictive markers, Prognostic markers

## Abstract

Computer-assisted reporting (CAR) tools were suggested to improve radiology report quality by context-sensitively recommending key imaging biomarkers. However, studies evaluating machine learning (ML) algorithms on cross-lingual ontological (RadLex) mappings for developing embedded CAR algorithms are lacking. Therefore, we compared ML algorithms developed on human expert-annotated features against those developed on fully automated cross-lingual (German to English) RadLex mappings using 206 CT reports of suspected stroke. Target label was whether the Alberta Stroke Programme Early CT Score (ASPECTS) should have been provided (yes/no:154/52). We focused on probabilistic outputs of ML-algorithms including tree-based methods, elastic net, support vector machines (SVMs) and fastText (linear classifier), which were evaluated in the same 5 × fivefold nested cross-validation framework. This allowed for model stacking and classifier rankings. Performance was evaluated using calibration metrics (AUC, brier score, log loss) and -plots. Contextual ML-based assistance recommending ASPECTS was feasible. SVMs showed the highest accuracies both on human-extracted- (87%) and RadLex features (findings:82.5%; impressions:85.4%). FastText achieved the highest accuracy (89.3%) and AUC (92%) on impressions. Boosted trees fitted on findings had the best calibration profile. Our approach provides guidance for choosing ML classifiers for CAR tools in fully automated and language-agnostic fashion using bag-of-RadLex terms on limited expert-labelled training data.

## Introduction

There are no studies available that evaluate machine learning (ML) algorithms on cross-lingual RadLex mappings to provide guidance when developing context-sensitive radiological reporting tools. Therefore, the goal of our study was to compare the performance of ML algorithms developed on features extracted by human experts against those developed on fully automated cross-lingual RadLex mappings of German radiological reports to English^1^, in order to assist radiologists in providing key imaging biomarkers such as The Alberta Stroke Programme Early CT Score (APECTS)^2^. We show that this fully automated RadLex-based approach is highly accurate even if the ML models were trained on limited and imbalanced expert labelled data sets^3–6^. Hence, this work provides a valuable blueprint for developing ML-based embedded applications for context-sensitive computer-assisted reporting (CAR) tools^7–10^.

RadLex is a comprehensive hierarchical lexicon of radiology terms that can be utilized in reporting, decision support and data mining^3^. RadLex is freely available (v.4.0, http://radlex.org/) from the Radiological Society of North America (RSNA). It provides the foundation for further ontologies and procedural data bases such as the LOINC/RSNA Radiology Playbook^11^ or Common Data Elements (CDE; RadElement; https://www.radelement.org/^12^. The official translation of RadLex to German by the German Society of Radiology (DRG) was made public in January 2018 and contained over 45,000 concepts.

ASPECTS was chosen for this study as a key radiological biomarker, as it is widely used in neurological emergencies to assess the extent of early ischemic changes on pretreatment non-contrast CT studies of the brain in patients with acute ischemic stroke of the anterior circulation^2^. It proved to be a robust and highly significant independent imaging biomarker to select patients for neurointerventional procedures^13^. Radiological textual metadata is of crucial importance when selecting patient cohorts for clinical trials or extracting their imaging retrospectively to develop applications using artificial intelligence (AI)^14–17^. Hence, it is in the best interest of radiologists to report key radiological biomarkers like ASPECTS or other scoring systems to optimize downstream analytics and software development^18,19^. Nonetheless, these key predictors are frequently missing from radiological reports as their overwhelming majority is still created as conventional narrative “free-text”^1,20,21^. In this work, we aim to provide blueprints for creating ML-based CAR tools using a domain-specific ontology to help radiologists improve the content of key biomarkers without disrupting their preferred “free-text” reporting workflow.

**Figure 1:**
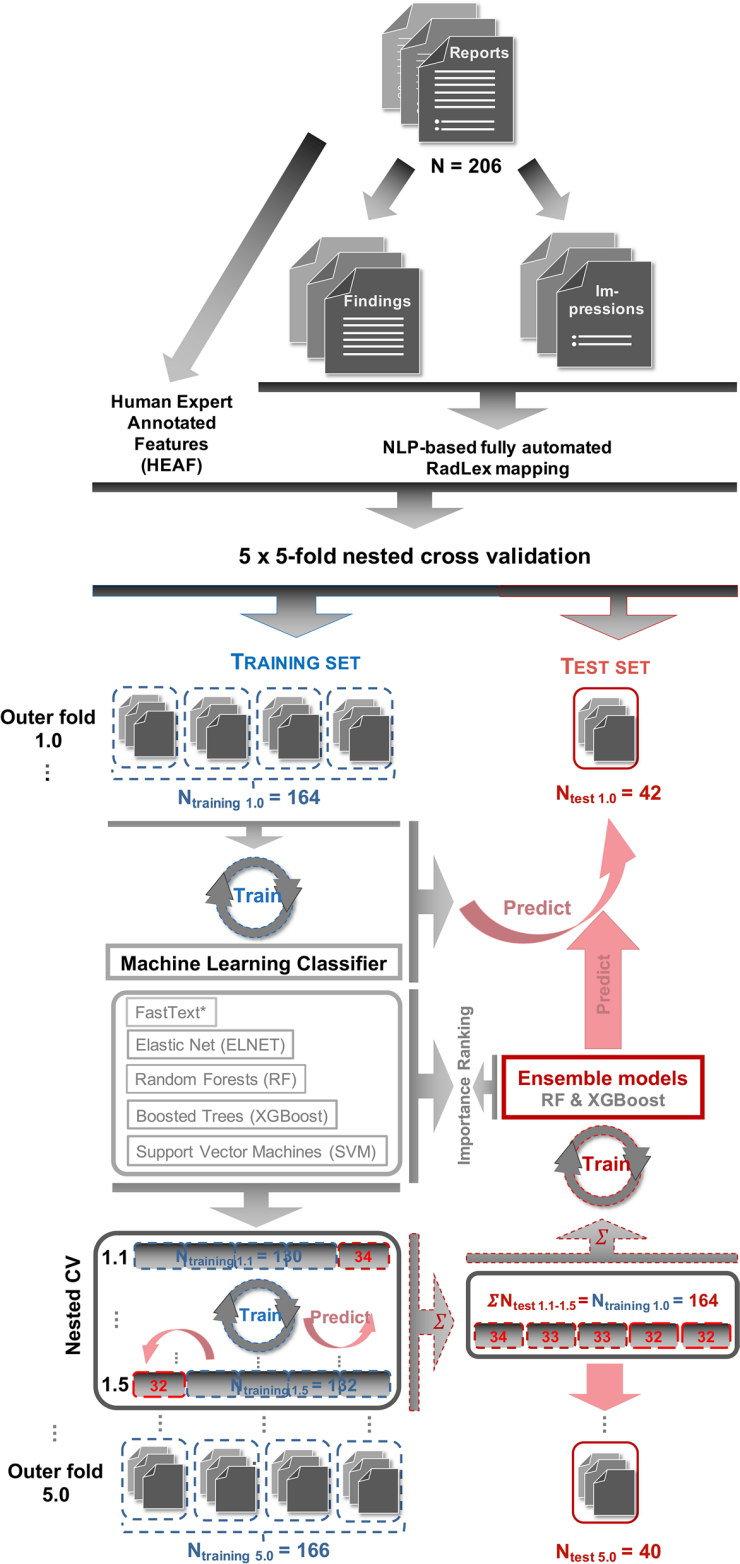
The 5 × fivefold nested cross validation setup, which was used to evaluate all machine learning (ML) algorithms and to train the second layer model as a meta/ensemble-learner on top of the combined predictions of these base ML classifiers. Human experts had access to both the findings and impression sections as well as the clinical question field of the reports to generate target labels ASPECTS recommended “yes” (n = 154) vs. “no” (n = 52) and to extract clinico-radiologically relevant features (HEAF). The findings and the impressions were each passed through a fully automated cross-lingual (German-English) natural language processing (NLP) pipeline to generating RadLex mappings. The pipeline can be accessed at https://mmatt.shinyapps.io/rasp/. In order to prevent information leakage, the second layer meta/ensemble models (random forests [RF] and boosted trees [XGBoost]) were trained on the combined inner fold test (i.e. sum of nested validation ΣN_test_1.1–1.5_) sets. These second layer models were used to derive objective importance rankings of the individual ML classifiers. To ensure direct comparability between the investigated ML-algorithms, the data partitioning was identical (i.e. each model was trained and fitted on the very same subsamples of the data). However, fastText was fitted directly on German report texts (*), whereas other ML-algorithms were fitted on both HEAF and NLP-based RadLex mappings. The final performance measure of the classifiers was calculated as the fivefold cross-validated average on the outer folds (see Tables 1, 2 and 3).

ML methods have been introduced as powerful computer-aided diagnostic (CAD) tools^9,15,22^ in medical image analysis and in radiological reporting^23,24^. Recently, complex deep transformer-based language models (TLM) are becoming the state-of-the-art (SOTA) in natural language processing (NLP)^25–29^. However, these models need considerable amount of general and domain specific corpora for training, which are scarce for languages other than English, particularly in the medical domain where creating expert-labelled high-quality training data is extremely resource intensive^30–33^. Despite achieving SOTA on certain classification tasks, TLMs represent black box methods and show susceptibility to subtle perturbances^31,32^. Additionally, TLMs are seldom compared to baseline information retrieval methods such as shallow ML algorithms or linear classifiers (fastText) developed on bag-of-words (BOW)^34–36^. Therefore, we performed comprehensive analyses using an ensemble learning framework (Fig. 1) that combined well-established ML algorithms as base classifiers including random forests (RF)^37^, regularized logistic regression (ELNET)^38,39^, support vector machines (SVM)^40^ and classification- (CART)^41^ and boosted trees (XGBoost)^42^ as well as fastText^36^ on German computed tomography (CT) reports with suspected stroke and on their cross-lingual English RadLex mappings using NLP^43^.

Our goal was to evaluate a flexible open-source pipeline to swiftly develop robust ML classifiers for CAR tools in a language-agnostic fashion by using cross-lingual bag-of-RadLex mappings on limited expert labelled training data. We aimed to demonstrate the feasibility of our approach by automatically developing production-ready ASPECTS classifiers for CT stroke workups (“MyReportCheck”, Suppl. Fig. S1 online) and compare its performance to ML models that were developed on human expert annotations.

## Results

### Inter-rater reliability of human experts

Providing ASPECTS in the report would have been recommended by R1 in 156 (75.7%), by R2 in 154 (74.8%) and by R3 in 155/206 (75.2%) of the cases. The overall agreement between the three readers for “ASPECTS recommended” was kappa_Light_ = 0.747 (n = 206, z = 4.6, p = 4.3 $\times$ 10^–6^). The pairwise Cohen’s kappa between R1 and R2 was 0.635 (p < 2 $\times$ 10^–16^), which corresponded to 86.4% agreement. Between R1 and R3 it was 0.62 (p < 2 $\times$ 10^–16^) corresponding to 85.9% agreement. Ratings of two (R2 and R3) experienced readers showed an almost perfect alignment kappa = 0.987 (p < 2 $\times$ 10^–16^) with 99.5% overall agreement.

### Reliability between automated RadLex mappings and expert-annotated labels

In this random subsample, which represents a robust cross-section of the daily praxis, ASPECTS was reported extremely rarely in 4/206 (1.9%). Three of which occurred both in the findings and impressions (3/4, 75%) section and one of which was only reported in the impression (1/4, 25%). The RASP tool correctly annotated all ASPECTS-negative (203/203) and ASPECTS-positive (3/3) finding sections. In the impressions, it misclassified one ASPECTS-positive (1/4, 25%) report as negative (1/206, 0.49%).

### Performance of machine learning algorithms developed on human expert-annotated features (HEAF)

CART demonstrated a fivefold CV accuracy of 73.3% with the worst 63% AUC, BS (0.37) and LL (0.87) values among the tested ML-classifiers (Table 1).

**Table 1:** Summary table of performance measures of the investigated ML algorithms developed on human expert-annotated features (HEAF).

Report section	Method	ML Classifier	HEAF feature space	Rank	Software	Optimized metric	Tested hyperparameter space	Selected number of features or hyperparameter settings on outer fold 1.0–5.0	Accuracy^#^ [min–max; %]	ME	AUC	BS	LL
Human Expert-Annotated Features (HEAF)	CART	CT	p = 28 (all)		rpart [R]	ACC	rpart.control = default; cp = 0.01 no optimization (no pruning)	28	73.3 [66.7–79.2]	0.27	0.63	0.37	0.87
	vRF	RF	p = 28 (all)	4	randomForest [R]	ME	ntree = 500, mtry = 5, p_varsel_ = 28	28	81.5 [73.8–92.7]	0.18	0.82	0.27	0.44
	vRF	RF	p = 28 (all)		randomForest[R]	ME	ntree = 500, mtry = 5, p_varsel_ = 9	9	71.0 [59.5–82.9]	0.29	0.69	0.37	0.56
	vRF	RF	p = 28 (all)		randomForest[R]	ME	ntree = 500, mtry = 5, p_varsel_ = 5	5	75.2 [68.3–83.3]	0.25	0.69	0.36	0.54
	tRF_BS_	RF	p = 28 (all)	2	randomForest[R]	BS	ntree = [100, 200, 300, … , 900, 1000]	28, 14, 14, 14, 14	83.1 [76.2–90.2]	0.17	0.81	0.27	0.44
	tRF_ME_	RF	p = 28 (all)		randomForest[R]	ME	mtry = [3, 4, 5, 6, 7]	28, 28, 14, 5, 14	79.6 [68.3–90.2]	0.20	0.79	0.29	0.46
	tRF_LL_	RF	p = 28 (all)	2	randomForest[R]	LL	p_varsel_ = [3, 5, 10, 14, 20, 25, 28]	25, 14, 14, 14, 14	83.1 [76.2–90.2]	0.17	0.81	0.27	0.44
	ELNET	ELNET	p = 28 (all)	3	glmnet[R]	ME	α = [0, 0.1, 0.2, … , 0.8, 0.9, 1] λ = tenfold CV with default hot-start	α = [0.1, 0.8, 0, 1, 0.1] λ = [0.195, 0.0688, 0.208, 0.0301, 0.1632]	82.0 [78.6–85.4]	0.18	0.79	0.27	0.43
	SVM-LK	SVM	p = 28 (all)	1	e1071[R]	ME	C = [0.001, 0.01, 0.1, 1, 10, 100, 1000]	C = [1, 1, 100, 10, 10]	87.4 [82.9–90.2]	0.13	0.79	0.22	0.37
	XGBoost	BT	p = 28 (all)	5	xgboost[R]	ME	nrouds/ntree = 100,	nrouds = 100	80.6 [75.0–85.7]	0.19	0.70	0.30	0.48
							max_depth = [3, 5, 6, 8]	max_depth = [5, 3, 5, 8, 3]					
							eta = [0.1, 0.3]	eta = [0.1, 0.1, 0.1, 0.3, 0.1]					
							gamma = [0, 0.5, 1.0]	gamma = [0, 0.5, 1, 0.5, 1]					
							colsample_bytree = [0.1, 0.25, 0.5, 0.693 (ln2) ~ ^RF^, 1]	colsample_bytree = [1, 1, 0.5, 1, 0.5]					

Accuracy#: the averaged fivefold CV accuracy is calculated, ACC: accuracy, AUC: multiclass area under the ROC after Hand and Till (that can only be calculated if probabilities are scaled to 1), BS: Brier score, ME: misclassification error, LL: multiclass log loss, vRF and tRF: vanilla- and tuned random forests, ELNET: elastic net penalized multinomial logistic regression, SVM: support vector machines, LK: linear kernel SVM; XGBoost: extreme gradient boosting using trees as base learners, BT: boosted trees, CART: classification and regression trees; CT: classification tree; cp: complexity parameter used for CART node splitting (for this no optimization (pruning) was performed); ln(2) ~ ^RF^: column sampling (i.e. bootstrap) representing the settings equivalent to running RF in the xgboost library, [R]: R statistical software environment.

The default (“vanilla”) RF classifier fitted on the 28 HEAF achieved a fivefold CV accuracy of 81.5% with an AUC of 82% and corresponding BS and LL of 0.27 and 0.44, respectively (Table 1). Drastically reducing the feature space of vRF to only the nine (9/28: 32.1%) or five (5/28; 17.9%) most important predictors, had a comparably limited effect on the predictive performance of vRF: its accuracies decreased 12.8% and 7.7%, respectively; AUC decreased by ~ 16%; while BS (~ 37%) and LL (~ 27%) scores increased (Table 1).

Fine tuning the RF classifier using the BS (tRF_BS_) and LL (tRF_LL_) metrics slightly improved the overall accuracy without relevantly changing the calibration metrics of the vRF algorithm (Table 1). On the outer folds, both tRF_BS_ and tRF_LL_ limited the feature space similarly – to the 14 or 25–28 most important variables. Interestingly, ME-optimized RF (tRF_ME_) achieved a slightly worse overall performance profile. Notably, on the outer fold 4.0, it limited the feature space to only the five RadLex terms.

ELNET showed a similar performance profile to RFs when fitted on the 28 HEAF but it achieved a narrower fivefold CV confidence range of its accuracies (78–86%) while obtaining similar AUC, BS and LL scores (Table 1). The mixing parameter alpha (α) was chosen 3 out of 5 times to fit ridge (0) or ridge-like (0.1, 0.1) models while twice to fit lasso (1) or lasso-like (0.8) models on the outer folds.

On HEAF linear kernel SVMs (SVM-LK) achieved the highest fivefold CV accuracy (87.4%) and lowest BS (0.22) and LL (0.37) scores while obtaining a similar AUC of ~ 80% to other ML classifiers (Table 1). The tuning parameter of C was selected as 1 on two outer folds suggesting a larger margin for the separating hyperplane while larger values of 10 or 100 were selected on the remaining three outer folds, suggesting a smaller-margin classifier.

Boosted decision trees were similarly accurate (80.6%) like tuned RF and ELNET. Despite the detailed tuning grid, XGBoost had overall somewhat worse performance profile than the other investigated ML algorithms, particularly AUC was lower at 70% for which we do not have a clear explanation.

### Performance of machine learning algorithms developed on fully automated RadLex mappings

Directly applying a single classification tree (CART) without optimizing its tree complexity (i.e. no pruning) showed on the findings similar overall accuracy (77.2%) to vRF with similar AUC and BS (Table 2) but with worse LL metrics. On the impressions, however, CART was tied for the 3^rd^ best accuracy (85.0%) but still it showed low AUC (0.75) and high LL (0.58) values.

**Table 2:** Summary table of performance measures of the investigated ML algorithms on the NLP-annotated bag-of-RadLex features of the findings and impressions sections.

Report section	Method	ML Classifier	RadLex feature space	Rank	Software	Optimized metric	Tested hyperparameter space	Selected number of features or hyperparameter settings on outer fold 1.0–5.0	Accuracy^#^ [min–max; %]	ME	AUC	BS	LL
Findings	CART	CT	p = 907	5	rpart [R]	ACC	rpart.control = default; cp = 0.01	p = 907	77.2 [70.8–82.7]	0.23	0.74	0.32	0.66
	vRF	RF	p = 300 (us var.filt.)		randomForest [R]	ME	ntree = 500, mtry = 30, p_varsel_ = 200	p_varsel_ = 200	76.2 [71.4–85.4]	0.24	0.78	0.33	0.51
	vRF	RF	p = 907 (all)		randomForest[R]	ME	ntree = 500, mtry = 30, p_varsel_ = 200	p_varsel_ = 200	72.8 [67.5–78.6]	0.27	0.78	0.33	0.50
	vRF	RF	p = 907 (all)		randomForest[R]	ME	ntree = 500, mtry = 30, p_varsel_ = 20	p_varsel_ = 20	71.4 [62.5–75.6]	0.29	0.74	0.40	0.63
	tRF_BS_	RF	p = 907 (all)		randomForest[R]	BS	ntree = [200, 400, 600, … , 1400, 1600]	p_varsel_ = [500, 50, 100, 100, 50]	75.2 [71.4–81.0]	0.25	0.76	0.33	0.51
	tRF_ME_	RF	p = 907 (all)		randomForest[R]	ME	mtry = [20, 25, 30, 35, 40]	p_varsel_ = [907, 907, 907, 907, 907]	74.3 [67.5–83.3]	0.26	0.77	0.33	0.50
	tRF_LL_	RF	p = 907 (all)		randomForest[R]	LL	p_varsel_ = [10; 20; 50; 100; 200; 500; 907]	p_varsel_ = [50, 50, 50, 50]	75.7 [70.7–85.7]	0.24	0.77	0.33	0.52
	ELNET	ELNET	p = 907 (all)	4	glmnet[R]	ME	α = [0, 0.1, 0.2, …, 0.8, 0.9, 1] λ = tenfold CV with default hot-start	α = [0.2, 0.7, 0.9, 1, 0.1] λ = [0.2685, 0.134, 0.0793, 0.114, 0.397]	79.6 [76.2–82.9]	0.20	0.75	0.29	0.46
	SVM-LK	SVM	p = 907 (all)	3	e1071[R]	ME	C = [0.001, 0.01, 0.1, 1, 10]	C = [0.1, 0.1, 0.1, 0.1, 0.1]	82.5 [78.6–85.4]	0.18	0.80	0.27	0.43
	XGBoost	BT	p = 907 (all)	1	xgboost[R]	ME	nrouds/ntree = 100,	nrouds = 100	85.4 [80.9–90.2]	0.15	0.78	0.25	0.45
							max_depth = [3, 5, 6, 8]	max_depth = [5, 8, 5, 8, 3]					
							eta = [0.1, 0.3]	eta = [0.1, 0.1, 0.1, 0.3, 0.1]					
							gamma = [0, 0.5, 1.0]	gamma = [0, 0.5, 1, 0.5, 1]					
							colsample_bytree = [0.1, 0.25, 0.5, 0.693 (ln2) ~ ^RF^, 1]	colsample_bytree = [1, 1, 0.5, 1, 0.5]					
	fastText	linear	direct fit on text	2	Fasttext [Python]	ACC & LL	default	–	83.0 [81.0–85.4]	0.17	0.81	0.29	0.98
Impressions	CART	CT	p = 675	4	rpart [R]	ACC	rpart.control = default; cp = 0.01	p = 675	85.0 [79.3–89.5]	0.15	0.75	0.26	0.58
	vRF	RF	p = 300 (us var.filt.)		randomForest [R]	ME	ntree = 500, mtry = 26, p_varsel_ = 200	p_varsel_ = 200	83.0 [71.4–88.1]	0.17	0.87	0.25	0.39
	vRF	RF	p = 675 (all)		randomForest [R]	ME	ntree = 500, mtry = 26, p_varsel_ = 200	p_varsel_ = 200	82.5 [71.4–88.1]	0.17	0.87	0.25	0.39
	vRF	RF	p = 675 (all)		randomForest [R]	ME	ntree = 500, mtry = 26, p_varsel_ = 20	p_varsel_ = 20	78.2 [70–85.4]	0.22	0.81	0.30	0.49
	tRF_BS_	RF			randomForest [R]	BS	ntree = [200, 400, 600, …, 1400, 1600]	p_varsel_ = [200, 100, 200, 500, 200]	80.0 [69.0–87.8]	0.20	0.85	0.26	0.41
	tRF_ME_	RF	p = 675 (all)		randomForest [R]	ME	mtry = [21, 26, 31, 36, 41]	p_varsel_ = [200, 675, 200, 675, 500]	83.0 [69.0–90.5]	0.17	0.85	0.25	0.41
	tRF_LL_	RF			randomForest [R]	LL	p_varsel_ = [10; 20; 50; 100; 200; 500; 675]	p_varsel_ = [50, 100, 50, 500, 50]	79.6 [71.4–87.8]	0.20	0.84	0.27	0.42
							nodesize = [1; 2 (1%); 10 (5%)]						
	ELNET	ELNET	p = 675 (all)	3	Glmnet [R]	ME	α = [0, 0.1, 0.2, …, 0.8, 0.9, 1]	α = [0.9, 0.4, 1, 0, 0.9]	85.0 [82.9–88.1]	0.15	0.85	0.22	0.37
							λ = tenfold CV with default hot-start	λ = [0.056–2.01]					
	SVM-LK	SVM	p = 675 (all)	2	e1071 [R]	ME	C = [0.001, 0.01, 0.1, 1, 10]	C = [0.1, 0.1, 0.01, 0.1, 0.01]	85.4 [80.0–90.2]	0.15	0.86	0.21	0.36
	XGBoost	BT	p = 675 (all)	5	xgboost [R]	ME	nrouds/ntree = 100,	nrouds = 100	83.0 [71.4–90.2]	0.17	0.83	0.26	0.44
							max_depth = [3, 5, 6, 8]	max_depth = [5, 3, 6, 5, 6]					
							eta = [0.1, 0.3]	eta = [0.3, 0.3, 0.1, 0.1, 0.3]					
							gamma = [0, 0.5, 1.0]	gamma = [0, 0, 1, 0.5, 0.5]					
							colsample_bytree = [0.1, 0.25, 0.5, 0.693 (ln2) ~ ^RF^, 1.0]	colsample_bytree = [0.1, 0.25, 0.1, 1, 0.1]					
	fastText	linear	direct fit on text	1	Fasttext [Py]	ACC & LL	default	–	89.3 [832 .3–97.6]	0.11	0.92	0.18	0.55

Accuracy#: the averaged fivefold CV accuracy is calculated, ACC: accuracy, AUC: multiclass area under the ROC after Hand and Till (that can only be calculated if probabilities are scaled to 1), us var.filt: unsupervised variance filtering using p = 300 most variable RadLex terms -this step was previous of training to prevent information leakage, BS: Brier score, ME: misclassification error, LL: multiclass log loss, vRF and tRF: vanilla- and tuned random forests, ELNET: elastic net penalized multinomial logistic regression, SVM: support vector machines, LK: linear kernel SVM; XGBoost: extreme gradient boosting using trees as base learners, BT: boosted trees, CART: classification and regression trees; CT: classification tree; cp: complexity parameter used for CART node splitting (for this no optimization (pruning) was performed); ln(2) ~ ^RF^: column sampling (i.e. bootstrap) representing the settings equivalent to running RF in the xgboost library, [R]: R statistical software environment; [Py] Python v3.7 programming language.

As for RF, applying unsupervised variance filtering to select the top 33% most variable RadLex mappings of the findings sections, improved the fivefold CV accuracy of vRF by ~ 4.7%. In contrast, the same variance filtering on the impression sections did not relevantly (0.6%) improve vRF’s accuracy (Table 2). Tuned RF models were slightly more accurate than the default vRF, however, tuning did not improve much upon the remaining calibration metrics.

**Figure 2:**
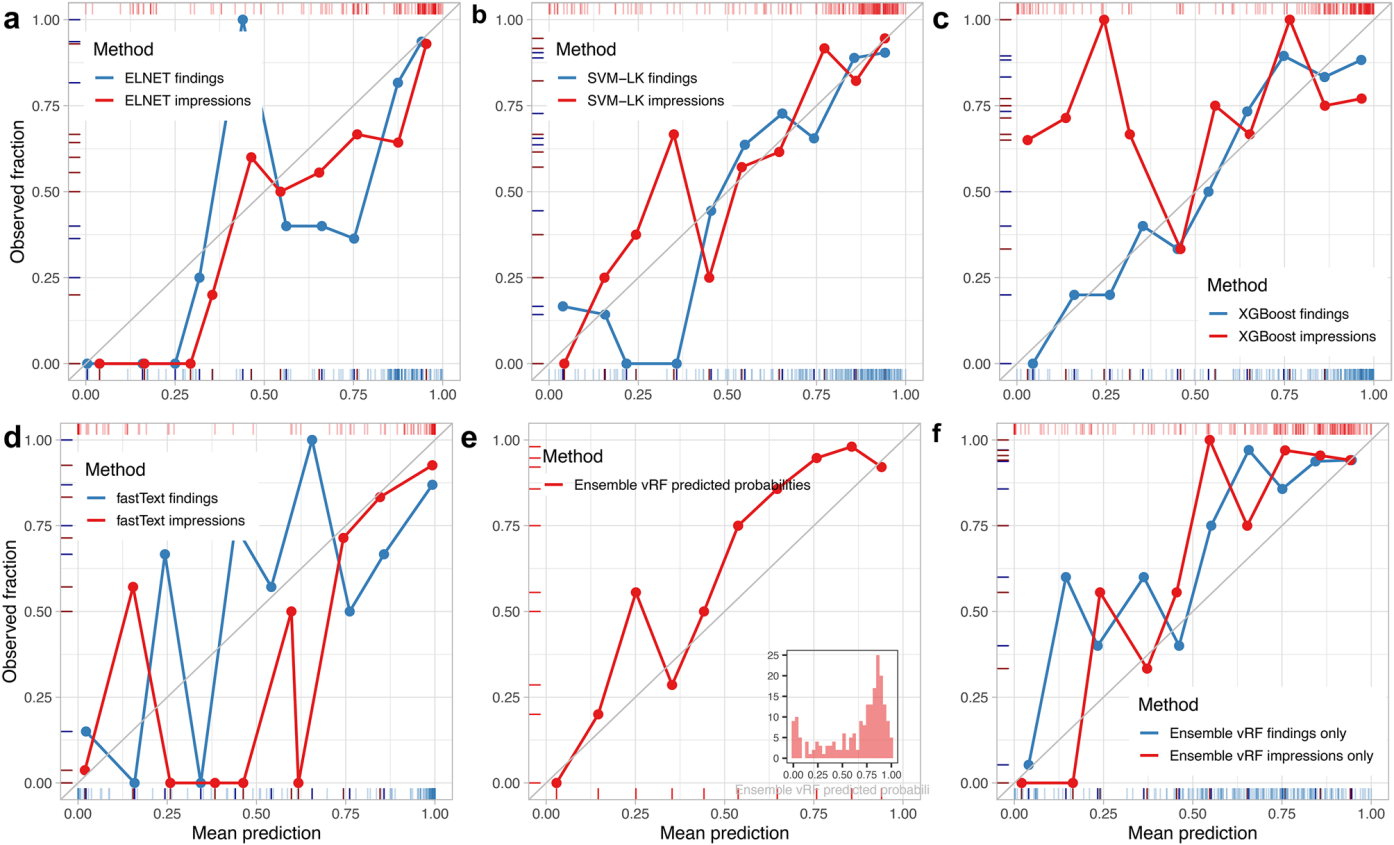
The calibration profiles of the best performing machine learning classifiers (**a**–**d**) fitted on the RadLex mappings and of the random forests meta/ensemble learner (**e**,**f**) fitted on the predicted probabilities of the ML-algorithms as features on all outer folds combined (N = 206). Probability estimates for each report by each ML classifier were recorded i.e. how likely it is that the predicted target label is “ASPECTS: yes”. The reliability of these predictions can be assessed visually on calibration plots. Calibration curves are created by grouping reports into discrete bins based on their assigned probability estimates by the ML-model. Thus, the probability space [0–1] gets discretized into bins (i.e. 0–0.1, 0.1–0.2, …, 0.8–0.9, 0.9–1.0; grey grid). The points represent the mean predicted probability (x-axis) and the observed fraction (y-axis) of true (“yes”) labels for the subset of reports falling in that respective range. For ideally calibrated models, the mean predicted probability and observed fraction should be identical within each bin, hence the calibration curve would lie on the diagonal (grey line). Rug plots (blue lines, findings; red lines, impressions) indicate the axis-values of the aforementioned aggregated bin measures (thick lines) and probability estimates of single reports (thin lines). ELNET (**a**) was more suitable for the impressions (red) particularly in the 0.50–0.75 range, corresponding to its top 3 ranked accuracy. Linear kernel SVMs (**b**) showed well-calibrated estimates for the 0.50–1.0 probability domain for both the findings (blue) and impressions (red). XGBoost (**c**) presented an almost ideal calibration curve on the findings (blue) while being the most accurate ML classifier (Table 2). FastText (**d**) achieved the highest overall accuracy when trained on the impressions (red) with partly well-calibrated estimates (0.75–1) but it was poorly calibrated on the findings (blue). The RF meta/ensemble learner (**e**) showed a reasonably well-calibrated profile when trained on probability outputs of all ML-algorithms (16 × ML models both findings and impressions; see Table 3). The histogram inset displays the bimodal distribution of its probability estimates. It showed (**f**) similar calibration profiles when trained either only on 8–8 ML model estimates of the findings (blue) or the impressions (red), respectively.

ELNET was the 3^rd^ best-performing ML algorithm on the RadLex features of the findings sections behind SVMs and XGBoost with similar BS and LL metrics but lower accuracy (p_Acc.vs.NIR_ = 0.061) and AUC (Table 2). On the impression, it achieved the second highest fivefold CV accuracy (85.0%; 95%CI: 79.3–89.5%; p_Acc.vs.NIR_ = 2.8 $\times$ 10^–4^) with corresponding second-best calibration profile (AUC: 86%; BS: 0.22; and LL: 037). On the outer folds of the impressions lasso or lasso-like settings (0.9–1) dominated the tuned α settings. ELNET had a better visual calibration profile on the impressions than on the findings (Fig. 2a).

Linear kernel SVMs (SVM-LK) were the only classifiers that performed in the top 2 on the RadLex feature spaces of both the findings (p_Acc.vs.NIR_ = 5.1 $\times$ 10^–3^) and impressions (p_Acc.vs.NIR_ = 1.4 $\times$ 10^–4^) sections (Table 2). SVM-LK had the highest AUC and lowest LL on the findings while on the impressions, it was overall the best-performing base ML-classifier. SVMs were comparably well-calibrated for both the findings and impressions, especially in the 0.5–1.0 probability domain (Fig. 2b).

XGBoost performed particularly well on the RadLex mappings of the findings – where the other ML algorithms (including fastText) struggled (Table 2). It showed the highest accuracy (p_Acc.vs.NIR_ = 1.4 $\times$ 10^–4^) and lowest BS with corresponding slightly worse AUC and LL metrics (than the runner-up SVM-LK). Nevertheless, it had the best overall visual calibration profile on the reliability diagrams for the whole probability domain (Fig. 2c). Compared to the findings, on the impressions XGBoost tuning implied a stronger subsampling of the features when constructing each tree, thereby strongly limiting the available predictor space. On the impressions, XGBoost performed similar to RF classifiers.

### Linear models (fastText) fitted directly on German report text

When directly fitting the findings sections of the reports, the fastText algorithm showed a fivefold CV accuracy of 83.0% (95%CI: 77.2–87.9%; p_Acc.vs.NIR_ = 0.0030) with sensitivity of 94.8%, and specificity of 48.1% (PPV 84.4%, NPV: 75.8%), which corresponded to 84.4% precision and 89.3% F1 score. It achieved comparable AUC (81.1%) and BS (0.29) to other shallow ML-models trained on RadLex mappings but showed markedly worse LL profile (0.98) suggesting “more certain” misclassifications.

FastText achieved the best results across all investigated ML algorithms fitted on the impressions sections of the reports. It showed a fivefold CV accuracy of 89.3% (95%CI: 84.3–93.2%; p_Acc.vs.NIR_ = 1.35 $\times$ 10^–7^) with a balanced accuracy of 82.0%. Its predictive profile was in the 87–97% range (sensitivity: 96.8%; specificity: 67.3%; PPV 89.8%, NPV: 87.5%) with precision of 89.8% and F1 score of 93.1%. Furthermore, it showed the highest AUC (91.7%) with lowest BS (0.18) but yet again somewhat worse LL (0.55) than the RadLex-based ML algorithms. FastText showed poor visual calibration profiles for both the findings and impressions in the lower probability domains (0–0.5), however it was almost ideally calibrated in the 0.75–1.0 domain of the impressions (Fig. 2d).

### Performance of the second layer meta/ensemble-learners

**Figure 3:**
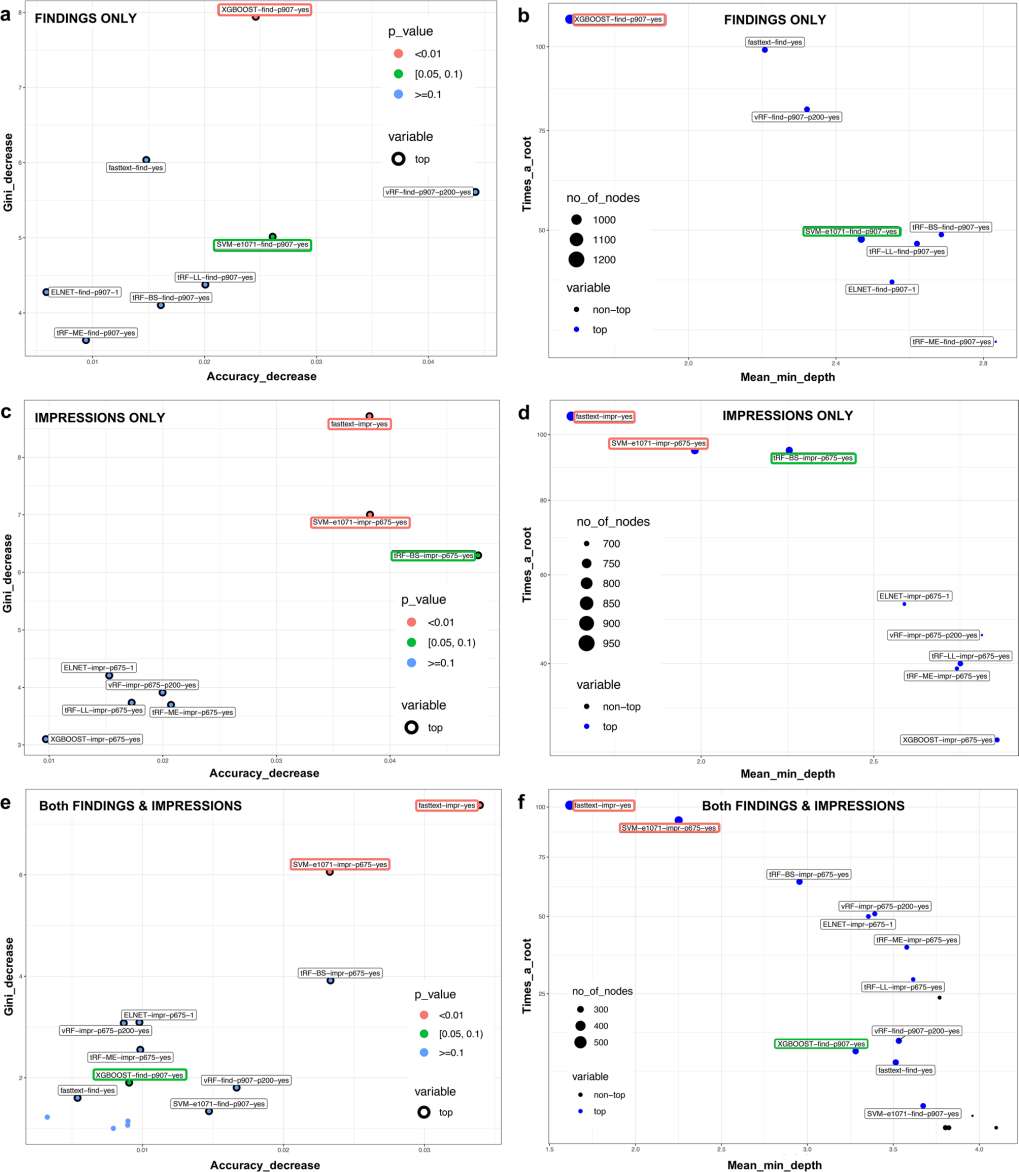
Two corresponding pairwise versions of multi-way importance plots of the investigated machine learning algorithms based on the random forests meta/ensemble learner when fitted on the probability estimates of the eight ML models as features (Table 3) based on the findings (**a,b**), impressions (**c,d**) and both (**e,f**) report sections. The axes on subplots (**a**,**c**,**e**) measure the prediction related relevance of a variable. Here, y-axes (Gini_decrease) display the Gini feature importance-based mean decrease in node impurity while the x-axes (Accuracy_decrease) show the more robust mean decrease in accuracy (type = 1) variable importance measure^6,62–64^. P-values (legend: red, green and blue patches and colored text brackets) were derived from a binomial distribution of the number of nodes split on the variable assuming random draws. On subplots (**b**,**d**,**f**), y-axes (Times_a_root) show the number of trees in which the root is split on that variable (i.e. ML classifier), whereas the x-axes (Mean_minimal_depth) show the mean depth of first split on the variable. Because these two measures are negatively associated, most important variables are located in the upper-left corner. Area of the points is proportional with the total number of nodes (no_of_nodes) in the forest that split on that variable and the points are blue if the variable was used as root (top). When ML classifiers trained only on the findings sections were fed to the RF ensemble (**a**), XGBoost (p < 0.01) was the only significant predictor while linear kernel SVM showed a weak trend (p < 0.1). Underscoring XGBoost’s importance (**b**), it was used in the most nodes and as root split. Among the models developed on the impressions (**c**), fastText (p < 0.01) was the most important predictor followed by SVM-LK (p < 0.01) while brier score-tuned RF (tRF-BS) showed a week trend (p < 0.1). FastText and SVM-LK (**d**) were the most relevant classifiers based on tree splitting measures. Likewise, when all 16 ML-models were combined (**e**), fastText (p < 0.01) and SVM-LK (p < 0.01) based on the impressions dominated the importance rankings, however, although less relevant findings-based XGBoost still achieved a weak trend (p < 0.1). Plots were created on the first outer fold test set (N_test.1.0_ = 42).

The second layer meta/ensemble RF learner, which was trained on predictions of the ML-classifiers of the findings sections, showed similar performance metrics (Table 3) as the top single ML-classifiers like SVM-LK, fastText and XGBoost (Table 2). Its accuracy was in the 77–88% 95%CI range (p_Acc.vs.NIR_ = 1.8 $\times$ 10^–4^) with 89.6% sensitivity; 65.3% specificity; 88.5% PPV; and 68% NPV which corresponded to a precision of 88.5% and F1 score of 89.6%. SVM-LK was chosen twice as the most important classifier while vRF, ELNET and XGBoost were each selected once on the five other folds (Fig. 3a,d).

**Table 3:** Summary table of performance measures of the second layer meta/ensemble learners (random forests and boosted trees) combining the predictions of all RadLex-based ML base classifiers from the findings and impression sections.

Ensemble ML-algorithm	Classifiers	Number of features (ML-model outputs)	Most important ML-classifiers/outer fold	Optimized metric	Hyperparameters	Selected number of features or hyperparameter settings on outer fold 1.0–5.0	Accuracy^#^ [95%CI]	ME	AUC	BS	LL
vRF	vRF	8 × ML-models (findings)	Top 1:	ME	ntree = 500, mtry = 2, p_varsel_ = 8	p_varsel_ = 8	83.5 [77.7–88.3]	0.17	0.83	0.29	0.47
	tRF_BS_,		vRF-find 1/5								
	tRF_ME_,		SVM-find 2/5								
	tRF_LL_,		ELNET-find 1/5								
	ELNET,		XGBoost 1/5								
	SVM-LK, XGBoost, fastText		Top 2:								
			XGBoost-find 1/5								
			tRF-ME-find 2/5								
			fasstext-find 1/5								
			ELNET-find 1/5								
vRF	vRF	8 × ML-models (impressions)	Top 1:	ME	ntree = 500, mtry = 2, p_varsel_ = 8	p_varsel_ = 8	89.3 [84.3–93.2]	0.11	0.90	0.19	0.34
	tRF_BS_,		fasstext-impr 5/5								
	tRF_ME_,		Top 2:								
	tRF_LL_,		svm-impr 1/5								
	ELNET,		XGBoost-impr 2/5								
	SVM-LK, XGBoost, fastText		tRF-BS-impr 1/5								
			ELNET-impr 1/5								
vRF	vRF	16 × ML-models	Top 1:	ME	ntree = 500, mtry = 4, p_varsel_ = 16	p_varsel_ = 16	88.8 [83.7–92.8]	0.11	0.90	0.20	0.36
	tRF_BS_,	(8 × findings &	fasstext-impr 5/5								
	tRF_ME_,	8 × impressions)	Top 2:								
	tRF_LL_,		svm-impr 3/5								
	ELNET,		tRF-BS-impr 1/5								
	SVM-LK, XGBoost, fastText		ELNET-impr 1/5								
XGBoost	vRF	16 × ML-models	Top 1:	ME	nrouds/ntree = [5, 10, 25, 50, 75, 100]	nrounds = [75, 5, 75, 5, 10]	87.4 [82.0–91.6]	0.13	0.87	0.30	0.46
	tRF_BS_,	(findings & impressions)	fasstext-impr 3/5		max_depth = [3, 5, 6, 8]	max_depth = [3, 6, 5, 3, 5]					
	tRF_ME_,		svm-impr 2/5		eta = [0.01, 0.1, 0.3]	eta = [0.3, 0.01, 0.1, 0.01, 0.1] gamma = [1, 0.01, 0.1, 0, 0.5] colsample_bytree = [0.1, 0.5, ln2^~RF^, 0.1, 0.25]					
	tRF_LL_,		Top 2:		gamma = [0, 0.001, 0.01, 0.1, 0.5, 1]						
	ELNET,		fasstext-impr 2/5		colsample_bytree = [0.1, 0.25, 0.5, 0.693 (ln2) ~ ^RF^, 1.0],						
	SVM-LK, XGBoost, fastText		tRF-BS-impr 2/5		min_child_weight = 1,						
			svm-impr 1/5		subsample = 1						

AUC: multiclass area under the ROC after Hand and Till (that can only be calculated if probabilities are scaled to 1), us var.filt: unsupervised variance filtering using p = 300 most variable RadLex terms -this step was previous of training to prevent information leakage, BS: Brier score, ME: misclassification error, LL: multiclass log loss, vRF and tRF: vanilla- and tuned random forests, ELNET: elastic net penalized multinomial logistic regression, SVM: support vector machines, LK: linear kernel SVM, n.SV: number of support vectors; XGBoost: extreme gradient boosting using trees as base learners, BT: boosted trees.

The fivefold CV accuracy (89.3%) of the ensemble RF (Table 3), when using only the ML-models of the impressions as input features, was identical to the best predictor (fastText). But the 95% confidence interval got narrower and the LL score got considerably reduced (by 38%). This solely impressions-based ensemble achieved the following metrics: sensitivity 92.2%; specificity 80.8%; PPV 93.4%, NPV 77.8% with corresponding precision of 93.4% and F1 score of 92.8%. FastText was chosen as the most important predictor for all outer fold test sets while as top 2^nd^ predictor XGBoost was chosen twice; ELNET, SVM-LK and tRF_BS_ were each selected once, respectively (Table 3; Fig. 3b,e).

When the ML-classifier predictions of both the findings and impressions were the combined input for the second layer RF model, its accuracy, BS and LL slightly got worse (5–6%). The confusion matrix derivates were as follows: sensitivity 91.6%; specificity 80.8%; PPV 93.4%, NPV 76.4% with corresponding precision of 93.4% and F1 score of 92.5%. The variable importance rankings were dominated by ML-classifiers developed on the impression sections (Table 3; Fig. 3c,f). The visual calibration profile of the RF ensemble developed on all ML-models (both findings and impressions; p = 16) are presented in (Fig. 2e,f).

On this same combined feature space (p = 16), the second layer XGBoost ensemble showed a slightly reduced accuracy and worse calibration profiles than the RF ensemble (Table 3). Its predictive profile was in the 82–92% range (p_Acc.vs.NIR_ = 6 $\times$ 10^–6^; sensitivity: 93.5%; specificity: 69.2%; PPV 90.0%, NPV: 78.3%) with precision of 90% and F1 score of 91.7%. XGBoost selected fastText impressions 3 × and SVM impressions 2 × out of 5 on the outer folds as the most important variable based on the gain metric.

## Discussion

In this work, we present a resource effective approach to develop production-ready embedded ML models for CAR tools, in order to assist radiologists in providing clinically relevant key biomarkers^9,20,44,45^. To our knowledge, this is the first study that uses fully automated cross-lingual (German to English) RadLex mappings-based machine learning to improve radiological reports by suggesting the key predictor ASPECTS in CT stroke workups. We demonstrated the feasibility of our automated RadLex framework (“MyReportCheck”, Supplementary Fig. S1 online) by comparing it to ML classifiers developed on human expert annotations. Furthermore, our ensemble learning setup provides objective rankings and a generalizable blueprint for choosing ML algorithms when developing classifiers for similar context-sensitive recommendation tasks^44,46^.

Although reporting templates have been developed to promote and standardize the best practice of radiological reporting^47–49^, the majority of radiology reports are still created in free-text format^50,51^. This limits the use of radiology reports in clinical research and algorithm development^45,49,51^. To overcome this, NLP pipelines including ML proved to be effective to annotate and to extract recommendations from reports^51,52^. Nonetheless, studies dealing with ML algorithm development particularly for real-time context-sensitive assistance of radiologists while writing reports are scarce^46,53^. Therefore, in this work, we focused on comprehensive and objective comparison of ML algorithms to provide technical guidance for developing these algorithms on limited (non-English) training data. For this, we have put an emphasis on the probabilistic evaluation and ranking of ML classifiers. This is less relevant for biomarker CAR recommendation systems but crucial for automated inference systems for scores such as BI-RADS^54^ or PI-RADS^18^.

We used a commercially available NLP pipeline that implements a common approach^8,51^ comprised of cleansing, contextualization and concept recognition as well as negation detection trained explicitly for German and English RadLex mappings^1,43^. This fully automated approach to generate bag-of-RadLex mappings is advantageous compared to standard BOW^35^ approaches, as it already captures domain-specific knowledge including negation and affirmation^3^. Mikolov et al. proposed word2vec to create semantic word embeddings, which gained popularity in the field of radiology^5,55^. However, word2vec struggles to properly handle out-of-vocabulary words^56,57^. Thus, it needs to be combined with radiology domain-specific mappings. In contrast, our approach directly generates bag-of-RadLex terms for each report. We then combine all binary RadLex term occurrences in our corpus (separately for findings and impressions) to generate the RadLex-DTMs. Therefore, our pipeline is also more robust for new or missing words e.g. if a new report does not contain certain terms (present in the training corpus), these can be easily substituted with 0 or new terms can be added to the DTM and the ML classifier can be swiftly retrained. This commercial NLP-based RadLex-mapping pipeline for creating DTMs is free for research purposes and can be easily utilized through our Shiny application.

Similar to previous studies^47,51^, we included all hierarchical parent and child elements of the tree structure of RadLex concepts as a flattened feature space and let the ML classifiers select subgroups of terms relevant to the classification task automatically during training. For a similar domain-specific semantic-dictionary mapping, as part of their hybrid word embedding model, Banerjee et al. created a custom ontology crawler that identified key terms for pulmonary embolism^57^. Another approach by Percha et al. included only partial flattening of RadLex. They selected the eight most frequent parent categories that were used to learn word and RadLex term vector representations for automatically expanding ontologies^5^. We have also found that certain key terms are missing from RadLex and manually extended it. Other approaches to mitigate this problem and to increase interoperability, aim to combine multiple (both radiology-specific and general medical) ontologies or procedural databases such as RadLex, LOINC/RSNA playbook, CDE from the RSNA and Systematized Nomenclature of Medicine Clinical Terms (SNOMED CT) as well as the International Classification of Diseases (v.10) Clinical Modification (ICD-10-CM)^56,58–60^.

All investigated ML algorithms were “CPU only” thereby imposing minimal hardware requirements and being quick both at train and test time^36^. These ML models have proven to be effective on both text classification^8,34,36^ and other high-dimensional medical problems including high-throughput genomic microarray data^6,61^. Additionally, we implemented a nested CV learning framework in order to objectively assess the importance of each ML base classifier and report section (i.e. findings and impressions) based on their probability estimates of recommending ASPECTS^6^. Zinov et al. also used a probabilistic ensemble learning setup to match lung nodule imaging features to text^53^. It is of note that there is multicollinearity both on the level of RadLex mappings when training ML base classifiers and when combining the probability estimates of these ML classifiers on the second layer meta/ensemble-learner level. Default settings of RF (both in Python and R) are less robust for these scenarios due to the dilution of true features^6,62–64^. To counter act dilution, we used the permutation-based importance (type = 1) without scaling for all RF models, which were suggested as the most robust settings in^6,63,64^. In contrast, boosted trees by design are less susceptible to correlation of features^42,65^. The performance of the investigated ML algorithms is differently sensitive to the number of features^6,61^. Based on results by limiting the feature space with unsupervised variance filtering, we suggest using all annotated RadLex features as input and treating the number of features (p) as a tuning parameter during ML-algorithm training to achieve the best possible accuracies.

ML models developed on HEAF were similarly accurate (87%) to those developed on fully automated cross-lingual RadLex mappings (~ 85%), although the latter models had substantially better calibration profiles (especially AUC and BS). This corresponded to results by Tan et al. on lumbar spine imaging when comparing rule-based methods to ML models^66^. On the more heterogeneous and larger RadLex feature space of the findings sections, most ML models including fastText struggled but XGBoost performed best with an almost ideal calibration profile among all models (including those developed on the impressions). As impressions are expert-created condensed extracts of the most relevant information, ML performed substantially better (all > 80%). Accordingly, both RF and XGBoost meta/ensemble learners favored ML models that were developed on the impressions particularly fastText, SVM-LK and BS-tuned RF. These second layer meta/ensemble models achieved precision of 90–93%, recall: 92–94% and F1 score: 91–93%, which was well in line with the performance of information extraction model by Hassanpour et al. on a similarly sized (n = 150) test set of multi-institutional chest CT reports^51^.

The advantage of RadLex-based ML models compared to fastText is that they contain anatomical concepts and we can directly access negation information providing human interpretable explanation of the model. For fastText, such concepts are not necessarily learnable from limited training data or for more complex decision support scenarios other than ASPECTS. This was also supported by the fact that, despite being a baseline model, single CART performed remarkable well on the impressions implying that recommending ASPECTS is a less complex decision task.

The present study has certain limitations as it was a single-center, retrospective cross-sectional study of limited size. Nonetheless, we tried to create a representative cohort of the general daily praxis by selecting a stratified random sample of ~ 200 reports from ~ 4000 reports from a period of 4 years, which may robustly represent the general daily praxis. Our primary goal was to provide baseline performance metrics for well-established NLP and ML algorithms and linear classifiers with respect to radiology-specific biomarker (ASPECTS) recommendation tasks. Hence, there are natural extensions to our traditional methodology including the switch to well-known neural network architectures at the level of concept recognition to generate RadLex mappings^26,67^. Recently, DL methods are increasingly used for concept recognition tasks such as long short-term memory (LSTM) and variants of bidirectional recurrent neural networks (BiRNN) coupled with conditional random field (CRF) architectures^68,69^. DL models can also be used to create task-specific classifiers in an end-to-end manner (e.g., convolutional neural (CNN)^24^, RNN^54^ or LSTM networks^45,70^). However, fastText (with only a single hidden layer) has proven to be on a par with these more complex network architectures on several benchmarks^36^. Although incorporating pre-trained language-specific word representations into fastText was expected to improve its accuracy, we chose not to do so to allow for more direct performance comparisons with bag-of-RadLex-based ML classifiers^71^.

Utilizing large transformer architectures^25,27–29,72^ directly on German free-text reports would be a reasonable extension, however, sufficiently large non-English public radiology domain-specific corpora for transfer learning are lacking and the interpretability of TLMs is challenging^31^. Whether TLMs “truly learn” underlying concepts as a model of language or just extract spurious statistical correlations is a topic of active research^32,33^. Thus, our CT stroke corpus can facilitate benchmarking of such models for the German radiological domain^31,67,72^.

For recommending ASPECTS we used p_yes_ > 0.5 probability threshold. Optimizing this cutoff could further improve the performance metrics of the ML classifiers – for example by maximizing the Youden index^73^.

To counteract class imbalance, we also explored upsampling, downsampling, random over-sampling and synthetic minority over-sampling techniques (SMOTE)^74^, however, they did not improve the accuracy of ML classifiers on our data set (data not shown).

Regardless of these limitations, compared to text-based DL methods, our approach has some major advantages: i) building ML classifiers on top of cross-lingual RadLex mappings incorporates domain-specific knowledge thereby only requiring a limited amount of expert labeled data – for which simple class labels may be sufficient; ii) this approach can be easily adopted to any other language where RadLex was translated by the local radiological society; iii) an ultimate benefit of our methodology is that it allows for the instant interoperability between languages especially the direct transportability of any ML model created for biomarker recommendation or inference from one language to another. Furthermore, the investigated ML algorithms has been proven to be effective for high-dimensional multiclass classification problems in various scientific domains^6^, therefore, are expected to generalize well for other (more complex) radiological key biomarkers with multiple outputs (e.g., BI-RADS^54^, PI-RADS^18^). However, developing classifiers for biomarkers that describe more complicated pathophysiological processes or entities (than ASPECTS) will possibly require lager data sets.

In conclusion, we showed that expert-based key information extraction and fully-automated RadLex mapping-based machine learning is comparable and requires only a limited amount of expert-labeled training data – even for highly imbalanced classification tasks. We performed detailed comparative analyses of well-established ML algorithms and identified those, which are best suited for automated rule learning on bag-of-RadLex concepts (SVM, XGBoost and RF) and directly on German radiology report texts (fastText) through utilizing a nested CV learning framework. This work provides a generalizable probabilistic framework for developing embedded ML algorithms for CAR tools to context-sensitively suggest, not just ASPECTS but any required key biomarker information. Thereby improving report quality and facilitating cohort identification for downstream analyses.

## Methods

### Study cohort

The study was approved by the local ethics committee (Medical Ethics Commission II, Medical Faculty Mannheim, Heidelberg University, approval nr.: 2017-825R-MA). All methods were carried out in accordance with institutional guidelines and regulations. Written informed consents were waived by the ethics committee due to the retrospective nature of the analyses. In this single-center retrospective cohort study, consecutive (German) radiological reports of cranial CTs with suspected ischemic stroke or hemorrhage between 01/2015–12/2019 were retrieved from local RIS (Syngo, Siemens, Healthineers, Erlangen, Germany) that contained the following key words in the clinical < request reason > , < request comment > or < request technical note > fields: “stroke”, “time window for thrombolysis”, “wake up”, “ischemia” and their (mis)spelling variations. A total of 4022 reports fulfilled the above criteria. After data cleaning, which excluded cases with missing requesting department, 3997 reports remained. Next, we generated a stratified random subsample (n = 207, ~ 5.2%) based on age (binned into blocks of 10 years), sex (M|F), year (in which the imaging procedure was performed) and requesting department. During downstream analyses one report was removed because it contained only a reference to another procedure, leaving n = 206 for later analyses (Fig. 1). The extracted reports were all conventional free-texts and were signed off by senior radiologists with at least 4 years of experience in neuroradiology.

### Information extraction by human experts

Three independent readers (R1, experience 3yrs; R2, 7yrs; R3, 10yrs) assessed the clinical questions, referring departments, findings and impressions of the reports. For each report, all readers independently evaluated whether ASPECTS was provided in the report or should have been provided in the report text (necessary: 154, 74.7%; not meaningful: 52, 25.3%]). Further, the two senior experts (R2 and R3) manually extracted clinico-radiologically relevant key features in the context of whether reporting ASPECTS is sensible based on the presence (yes | no) of ischemia (separately for new infarct demarcation and/or chronic post-ischemic defects); bleeding (separately for each of the following entities: intracerebral hemorrhage (ICH), epi- (EDH), subdural hematoma (SDH), subarachnoid hemorrhage (SAH)); tumor; procedures including CT-angiography (CTA) or CT-perfusion (CTP); whether cerebral aneurysms or arteriovenous malformations (AVM) were detected; previous neurosurgical (clipping, tumor resection) or neurointerventional procedures (coiling); and previous imaging (within the last 1–3 days)^75,76^. These human expert-annotated features (HEAF) were extracted concurrently from both the finding and impression sections and selected in accordance with national and international guidelines for diagnosing acute cerebrovascular diseases^75,76^. HEAFs were used as input for ML algorithm development (Table 1). The feature matrix is available as supplementary data (heaf.csv) or GitHub download (https://github.com/mematt/ml4RadLexCAD/data).

### RadLex mapping pipeline

Both the findings and impression sections of each German report (n = 206) were mapped to English RadLex terms using a proprietary NLP tool, the Healthcare Analytics Services (HAS) by Empolis Information Management GmbH (Kaiserslautern, Germany; https://www.empolis.com/en/). As previously described^1,43^, HAS implements a common NLP pipeline consisting of cleansing (e.g., replacement of abbreviations), contextualization (e.g. into segments "clinical information", "findings", and "conclusion"), concept recognition using RadLex, and negation detection ("affirmed", "negated", and "speculated")^77^. HAS was pre-trained on ~ 45 k German radiological reports^1,43^. For concept recognition, a full text index and morpho-syntactic operations such as tokenization, lemmatization, part of speech tagging, decompounding, noun phrase extraction and sentence detection were used. The full text index is an own implementation with features such as word/phrase search, spell check and ranking via similarity measures such as Levenshtein distance^78^ and BM25^79^. The index is populated with synonyms for all RadLex entities (both from the lexicon and by manual extensions), the morpho-syntactic operations are based on Rosette Base Linguistics (RBL) from Basis Technology (Cambridge, MA, USA; https://www.basistech.com/text-analytics/rosette/). For accuracy, RBL uses machine learning techniques such as perceptrons, support vector machines, and word embeddings. For negation detection, the NegEx algorithm was implemented in UIMA RUTA^77,80^. No further pre-processing steps of the text were done.

Our RadLex annotation and scoring pipeline (RASP), which utilizes the aforementioned HAS API, is freely available as a Shiny application at https://mmatt.shinyapps.io/rasp/^35^. We used RASP to generate the document (i.e. report RadLex) term matrix (DTM) of the complete data set over all reports (n = 206) both for the findings and impression sections, respectively. In the DTM, each report is represented as a vector (i.e. bag-of-)RadLex terms that occurred in the corpus^34,35^. All hierarchical parent and child categories of the identified RadLex terms were included as features and encoded in a binary fashion (0|1), whether the term was present or not. Other kinds of relationships such as “May_Cause” were disregarded. Further, each RadLex term (i.e. feature) was annotated with three levels of confirmation or confidence “affirmed”, “speculated”, “negated”, which was included in the feature name. Feature names were generated by combining the RadLex ID, preferred name of the term and the assigned confirmation level. This DTM provided the basis for fully automated RadLex-based ML algorithm development (Table 2). The report-RadLex term-matrices (i.e. DTMs) both for the findings and impression sections are available for direct download from our GitHub repository (https://github.com/mematt/ml4RadLexCAD/data) or as supplementary data (radlex-dtm-findings.csv and radlex-dtm-impressions.csv).

The performances of ML algorithms developed on these automated NLP-RadLex mappings were then compared to those ML algorithms that were developed on the features extracted by human experts (HEAF). It is of note, however, that in its current iteration (v4.0) RadLex does not contain certain key terms or concepts, one of which is ASPECTS. Although there is a CDE for ASPECTS classification (https://www.radelement.org/element/RDE173)^12^. Hence, extended IDs had to be created for such terms in the NLP annotation service, which are denoted as RadLex ID Extended (RIDE), for example ASPECTS = RIDE172 in the DTMs.

### Classifiers and feature importance

We performed extensive comparative analyses of well-established ML algorithms (base classifiers) to automatically learn rules required for ASPECTS reporting including single classification (and regression) trees (CART)^41^, random forests (RF)^37^, boosted decision trees (XGBoost)^42^, elastic net-penalized binomial regression (ELNET)^38,39^ and support vector machines (SVM)^40^. Single CART was used to represent the baseline ML algorithm. A CART has the advantage that human readers can more easily interpret it, however its estimates are much less robust than ensembles of trees like RF^41,65,81,82^. It is of note that RadLex mappings are inherently correlated features due to RadLex’s hierarchical design. This makes RF susceptible to miss the truly relevant terms and dilute the selected features^6,62–64^. Therefore, we used the most robust metric of permutation-based variable importance (type = 1) without scaling (scale = F) for all RF models^6,62–65^. Permutation-based variable importance quantifies the importance of a feature by defining a baseline accuracy (for classification tasks) when the initially trained RF model is fitted on the out-of-bag (OOB) samples^62,63^. Next, all values (observations) of a variable of interest (X_i_) are permuted in the OOB samples thereby breaking down any associations between X_i_ and the outcome. Then, the initial RF model (i.e. each individual tree in the forest) is refitted on this permuted OOB sample and the prediction accuracy is recalculated. The importance of a variable is the difference between the baseline and the drop in overall accuracy after permuting the values of X_i_. Notably, the RF classifier is not retrained after permutation, but the already trained baseline model is used to predict on the perturbed OOB sample. Consequently, calculating permutation-based importance metrics for several predictor variables is computationally more expensive than generating the mean decrease in impurity (Gini index) but also proved to be more robust^64,83,84^. It has also been shown that the raw (unscaled) permutation-based importance measures have better statistical properties^83^, although they are still potentially biased towards collinear features^84^. Therefore, we also compared RF to boosted trees, which are by design less susceptible to correlated features^42,65^. Importance ranking of boosted trees models (both at the annotated feature and meta-learner levels) were derived using the gain metric.

### Machine learning setup

Each ML algorithm was fitted to the i) human expert-annotated features (HEAF; Table 1) and to the ii) RadLex mapped DTMs both for the findings and impressions separately (Table 2).

Because the effort of manually annotating the data set is large, especially if multiple experts annotate the same reports, we built upon our previously open-sourced protocol of a fivefold nested cross-validation (CV) resampling scheme to have an objective and robust metric when comparing the performance of the investigated methods (Fig. 1). Nested CV schemes allow for the proper training of secondary (e.g. calibrator or ensemble) models, without allowing for information leakage (Fig. 1). To counter act the class imbalance (yes:no = 3:1) during CV-fold assignment (nfolds.RData), we performed stratified sampling. Also, RFs were downsampled to the minority class during training^62,85^.

In brief, the data set (n = 206) was divided into stratified subsamples (outer fold training [n_outer.train_ =  ~ 164–166] – test set pairs [n_outer.test_ = 40–42]) using fivefold cross-validation (Fig. 1; dashed blue and red boxes). Then, only the outer fold training sets were, yet again, subsampled using fivefold CV, in order to create the nested/inner fold (training [n_inner.train_ = 130–134] – test set pairs [n_inner.test_ = 32–34]; Fig. 1, nested CV). This was performed for both the findings and impressions sections using identical fold structures (Fig. 1).

Hyperparameter tuning (i.e. training) of the investigated ML algorithms (base classifier) was performed within an extra-nested CV loop on the outer- or inner fold training sets. All models were fitted to the same data structure. Also, random seeds were fixed across all ML algorithms, in order to ensure direct comparability of their performance measures. ML algorithm training was optimized using either accuracy, brier score or log loss, which is indicated along the tuning parameter settings in Tables 2 & 3. For all ML algorithms probability outputs were also recorded and used to measure AUC and to create calibration plots. The average fivefold CV model performances on the outer fold test sets are provided in Tables 1, 2 & 3.

We chose this nested CV setup to be able to use an independent second layer model. The rationale for this was to investigate whether using the probability outputs of the base ML classifiers as input features for a second layer ensemble model, it could improve the overall performance of suggesting ASPECTS; and to use this “meta/ensemble” learner to derive importance rankings of the investigated ML algorithms. Hence, we could objectively rank the ML algorithms in addition to comparing their performance metrics. Because these probability estimates represented highly correlated features, we chose RF and XGBoost as meta learners (as described above). RF and XGBoost were trained on the combined probability predictions (i.e. “ensemble”) of the base ML models (i.e. CART, RF, XGBoost, ELNET, SVM and fastText) on the respective nested/innerfold test sets (Fig. 1). Then, this tuned model was evaluated on the corresponding outer fold test set preventing any information leakage^6^. For RF ensemble, we have used mean decrease in accuracy without scaling that has been suggested as the most robust setting when fitting correlated features^6,62–64^. Importance ranking of boosted decision trees were generated by the gain metric^42^. Multi-way variable importance plots describing the RF meta learner (Fig. 3) were created using default settings of the “plot_multi_way_importance” function in the randomForestExplainer R package (v0.10.0.)^86^. Heretofore, we refer to second layer RF and XGBoost algorithms as meta/ensemble learners or models.

### Text classification directly on German report texts using fastText

We used the open-source, lightweight fastText library (v0.9.1; https://fasttext.cc/) to learn linear text classifiers for ASPECTS recommendations on our data set^36^. The German report texts (both findings and impression sections) were preprocessed by excluding the following special characters “([-.!?,'/()])”. It is of note that fastText was only trained “on-the-fly” in each resampling loop on the corresponding subset of ~ 130–165 reports and we did not utilize any pre-trained word vector model for German^71^. This approach ensured a more direct comparability with the ML-classifiers developed on bag-of-RadLex mappings. However, pre-trained word vector models for 157 languages, which were pre-trained on Common Crawl and Wikipedia by the fastText package authors are available for direct download (https://fasttext.cc/docs/en/crawl-vectors.html)71. We used the Python (v3.7) interface to fastText (https://github.com/facebookresearch/fastText/tree/master/python) on an Ubuntu 19.10 machine. FastText models were fitted both on the findings and impression sections respectively, using the same 5 × fivefold nested-CV scheme as for the other ML algorithms with similar extra-nested CV loop for training on the outer- or inner fold training sets. Class label predictions and probability outputs were recorded and evaluated in the same manner as the investigated ML algorithms developed on HEAF and RadLex mappings.

### Statistical analyses

All statistical analyses were performed using the R language and environment for statistical programming (R v3.6.2, R Core Team 2019, Vienna Austria). The Cohen’s kappa statistic was used to assess inter-rater agreement whether ASPECTS is recommended in a pairwise fashion for each of the two readers. To assess the overall agreement among the three readers, Fleiss’ and Light’s kappa was used.

Performance was evaluated using calibration metrics focusing on the probabilistic output of the ML base classifiers including the area under the ROC curve (AUC), brier score (BS) and log loss (LL) measures; and derivatives of the confusion matrix: sensitivity, specificity, positive- (PPV) and negative predictive value (NPV) as well as precision, recall and F1 scores. P-values (p_Acc.vs.NIR_) were provided to quantify the level of accuracy achieved by a ML classifier compared to the no-information rate (NIR) i.e. always predicting only the majority class (154/206, 74.8%). P-values < 0.05 were considered significant.

### Calibration plots

Calibration plots (or reliability diagrams) are useful graphical tools to visually assess the quality of the probability output of a classifier^87,88^. Custom functions are available on GitHub (https://github.com/mematt/ml4RadLexCAD/tree/master/calibrationplots) to generate calibration plots presented in Fig. 2. Briefly, for real-life problems the true conditional probabilities of target classes are often unknown, therefore the prediction space needs to be discretized into bins^88,89^. A common approach is to use ten bins (e.g., probability ranges: 0–0.1, 0.1–0.2, …, 0.9–1.0) and assign cases to the corresponding bin where their predicted probabilities by the respective ML classifier fall. Consequently, in each bin there is a distinct subset of the study cohort. For each bin the fraction of true positive cases in that subset (y-axis) is plotted against the mean of the predicted probabilities of the subset by the classifier (x-axis). Hence, the probability output of an ideally calibrated ML classifier would lie on the diagonal line^87,89^. For instance, if (hypothetically) ELNET estimated the predicted probability of “ASPECTS: yes” between 0.9–1.0 with mean ~ 0.9 for 10 of the reports based on RadLex mappings of their findings and impressions sections, respectively (Fig. 2a, x-axis) and if ELNET was well-calibrated, then the number of reports in which ASPECTS should be truly provided among these 10 reports, would ideally be 9. Hence, the observed fraction of such reports in the cohort (Fig. 2a, y-axis) would be (9/10 = 0.9) identical to the mean prediction^6,90^. The point coordinates representing the mean predicted probability by ELNET (Fig. 2a) and observed fraction in the cohort for this probability bin (0.9–1.0) were, indeed, both very close (red, impressions; blue, findings) and lied almost on the diagonal line^87,88^. Thus, ELNET was well-calibrated for this bin, but it was poorly calibrated (“unsure”) for the 0–0.25 or 0.5–0.75 ranges as the distance from the diagonal line was larger. Predictions based on the findings or impression varied substantially even with the same ML model (Fig. 2a–f).

## Supplementary Information

Supplementary Information 1.

Supplementary Information 2.

Supplementary Information 3.

Supplementary Information 4.

## Data Availability

Both the human expert annotated features (heaf.csv) and the fully automated NLP-based RadLex mappings (term-report-matrices) are provided in our GitHub repository (https://github.com/mematt/ml4RadLexCAD/). The RadLex annotation and scoring pipeline (RASP) is freely available for research purposes as Shiny application at www.mmatt.shinyapps.io/rasp . All tuned ML-model objects including the fold IDs for the 5 × fivefold stratified nested CV scheme (nfolds.RData) are provided on GitHub. Additionally, we provide R code for ML-model training and for generating calibration plots presented in Fig. 3.
