# Supplementary material for: Comparative analysis of machine learning algorithms for computer-assisted reporting based on fully automated cross-lingual RadLex mappings
Source: Sci Rep. 2021 Mar 9;11:5529. doi: 10.1038/s41598-021-85016-9 (PMC7970897; doi:10.1038/s41598-021-85016-9)
Supplement: Supplementary file 4 — Supplementary Information 4. [file 41598_2021_85016_MOESM4_ESM.docx]

**Supplementary materials**

**Supplementary Figure 1**

**Suppl. Figure S1** depicts the prototype graphical user interface of our context-sensitive CAR tool “MyReportCheck”, which utilizes our embedded machine learning framework on fully automated RadLex mappings for recommending ASPECTS during the reporting of neuroradiological emergencies. The software solution is available as service from Empolis Information Management GmbH (Kaiserslautern, Germany).
